# Physician-Authored Feedback in a Type 2 Diabetes Self-management App: Acceptability Study

**DOI:** 10.2196/31736

**Published:** 2022-05-10

**Authors:** Eden Potter, Frada Burstein, Daphne Flynn, In Dae Hwang, Tina Dinh, Tian Yu Goh, Mina Mohammad Ebrahim, Christopher Gilfillan

**Affiliations:** 1 Design Health Collab Monash Art, Design and Architecture Monash University Melbourne Australia; 2 Faculty of Information Technology Monash University Melbourne Australia; 3 Eastern Health Clinical School Faculty of Medicine, Nursing and Health Sciences Monash University Melbourne Australia

**Keywords:** mobile app, mobile apps, apps, mHealth, mobile health, smartphone, mobile phone, digital health, health, type 2 diabetes, diabetes mellitus, empirical test, activity, food consumption, daily feedback, behavior change

## Abstract

**Background:**

Type 2 diabetes (T2D) is increasingly prevalent in society, in part because of behavioral issues, with sedentary behavior, reduced exercise, and the consumption of foods with a high glycemic index being major contributors. There is evidence for the efficacy of mobile apps in promoting behavior change and lifestyle improvements in people with T2D. Many mobile phone apps help to monitor the condition of people with T2D and inform them about their health. Some of these digital interventions involve patients using apps on their own or in conjunction with health care professionals.

**Objective:**

This study aimed to test the acceptability of receiving app-based, daily physician feedback for patients with T2D that is informed by the continuous monitoring of their activity, food choices, and glucose profiles, with the aim of encouraging healthier behavior. The *GLOOK!* app was designed and developed by an academic research team and pilot-tested at an Australian public hospital.

**Methods:**

A total of 15 patients diagnosed with T2D wore a glucose monitor and an Apple Watch for 12 days. The uploaded data were integrated into the *GLOOK!* app on the patients’ smartphones, which also enabled the recording of activity and consumed food. A physician provided daily feedback to each individual through the app based on their data from each of the 12 days. At the beginning and end of the study, data were collected on vital signs, anthropometry, hemoglobin A_1c_ level, fructosamine level, and fasting lipids level. Participants were also interviewed at the beginning and end of the study to assess the acceptability of the intervention and its potential impact on promoting positive behavior change.

**Results:**

Over the 12 days of the study, there was a significant reduction of 0.22% (*P*=.004) in hemoglobin A_1c_ level. There were favorable changes in fructosamine and lipid fractions; however, none reached significance. There was also a fall of 0.65 kg in body weight and falls in blood pressure and pulse rate that did not reach significance. Patient feedback on the *GLOOK!* system was positive. Of the 15 participants, 13 (87%) were enthusiastic about continuing to use the app system if some usability and reliability aspects were improved. All participants regarded the personalized physician feedback as supportive and helpful in understanding their own health behavior. Of the 15 participants, 4 (27%) felt that using the system encouraged long-term behavior changes.

**Conclusions:**

A mobile app system that provides people with T2D daily, physician-generated, personalized feedback can produce favorable changes in glycemic and cardiovascular risk parameters—even in the short term—and encourage better self-management of their condition. Study participants found the experience of using the mobile app system acceptable and were motivated to establish longer-term lifestyle improvements through behavior changes.

## Introduction

### Background

Type 2 diabetes (T2D) is a widespread chronic health condition that is increasingly prevalent in society, in part because of people’s behavior. A lack of physical exercise and the consumption of foods with a high glycemic index are at the core of the current epidemic of obesity and increased risk of diabetes and consequent cardiovascular disease [1,2]. As T2D is the fastest growing chronic disease in Australia [3], there is a significant burden on the health system and on individuals themselves to manage their disease. Australian estimates put the prevalence of prediabetes at 10% (approximately 2 million people), with a conversion rate to diabetes of 2% to 3% per year. Known T2D affects 1.2 million Australians, with a further 500,000 undiagnosed cases, and health care costs are estimated at Aus $14.6 billion (US $10.9 billion) [4]. In addition, the high incidence of prediabetes amplifies this concern as these participants are destined to develop diabetes in the future and also intrinsically carry an increased cardiovascular risk. Current models of health care involve periodic reviews by health care professionals and delivery of education at a long interval of several months. This model often fails to provide sustained change, as multifaceted behavioral adjustments and commitment to self-care are required to achieve treatment goals [5]. We believe that more frequent and personalized feedback is likely to promote the motivation of patients with T2D to sustain positive behavior change by addressing their unmet need for self-care support [5,6].

The core activities in chronic disease self-management are medical management (medication and dietary advice adherence), management of necessary behavior changes, and managing emotions and feelings around coping with chronic diseases. This aspect of T2D treatment is relatively underdeveloped worldwide [7]. Improving systems for and providing active support with patient self-management can motivate sustained behavior change, reduce health complications, and reduce associated costs [6,7].

Several mobile health apps have been developed to help people with T2D self-monitor their condition and provide them with diabetes education and information. The increased use of health-related apps is partly because of their convenience, portability, and reach [3] and partly because of the high smartphone ownership; in 2021, almost 80% of Australians were estimated to be using smartphones [8]. Approximately 1800 of the >50,000 health care apps available on both the web-based app store and Google Play Store [9,10] were specifically for diabetes management [11], with diabetes being the primary chronic disease targeted by the mobile health industry, followed by asthma and depression [12]. Mobile app developers and publishers consider diabetes care in digital health as having the best market potential in any health field, with artificial intelligence (AI) being a major transformative force in the sector. In diabetes self-management apps, AI can be used to perform the tasks of advanced analytics, machine learning, and symbolic reasoning to support patient decision-making [13]. Currently, diabetes management apps offer a range of features such as blood glucose meter interconnectivity, real-time feedback, fitness tracking, diabetes education, psychosocial support, tracking of sugar and glucose levels and meal content, and recommendations on meal changes [11].

### Prior Work

Mobile phone interventions for diabetes self-management have been found to be a useful support in promoting health-related behavior changes. People tend to keep their phones with them constantly—even at night—thus providing an inexpensive, real-time delivery mechanism for health and behavioral support messaging [6]. Self-management apps for diabetes can help patients monitor their condition and provide input to self-education, complementing information about a more suitable diet [14]. Studies on the use of mobile apps for diabetes self-management suggest that useful features of apps include hemoglobin A_1c_ (HbA_1c_) tracking and monitoring of medication, meals and nutrition, physical activity, physical health, and mental well-being. Apps can deliver up-to-date diabetes education and patient reminders about taking medication and engaging in physical activity [15-18]. Some apps include *coaching* in the form of telemanagement and 2-way consultations with health care providers, who can remotely follow up and provide recommendations based on patient-generated health data collected by the diabetes management app and system [12].

More than 10 systematic literature reviews of studies on the use of mobile apps for self-management of diabetes have been published in the past 5 years [3,11,12,14-22]. Overall, 57 primary studies were included in this review. These studies found that using diabetes self-management apps can significantly improve the health outcomes of patients with T2D. In 18 of the 25 reviews, Greenwood et al [18] found that HbA_1c_ (average blood glucose) levels were significantly reduced through the use of technology-based self-management solutions. Randomized controlled trials on apps used specifically in T2D management have shown positive outcomes for app users, particularly in lowering HbA_1c_ levels and hypoglycemia [12]. In addition, when people with T2D are able to connect data on their monitored glucose levels with self-generated data from other health-related behaviors such as exercise, they are better informed and motivated to improve their self-care [11].

Core features in diabetes self-management apps vary widely among different apps [14,15], particularly in the extent to which these features are included. Studies have found that low-risk diabetes apps (those that offer education and health tracking rather than handling insulin dosing) are not regulated [14,20,21] and that mobile health apps lack evidence-based support when compared with clinical guidelines for disease management [12,16]. Regulation may improve app accuracy, information quality, and clinical validity, as well as enable patients to select the most suitable mobile app for their needs.

Previous studies on diabetes mobile app interventions have explored different types of feedback messaging based on several behavior change theories. These include targeting messages based on the patient’s disease stage within the transtheoretical model of behavior change [5] or using social cognitive theory and protection motivation theory [23]. Other mobile app message types are triggered by biometric and activity inputs, such as continuous glucose monitoring metrics, blood pressure levels, and data on activity levels and diet [24]. Baptista et al [11] suggested that advice conveyed by diabetes self-management apps that allow for reflection and interpretation, leading to specific and actionable recommendations, is the most useful for patients with diabetes. For example, receiving specific advice on how a meal could be healthier (Table 1) is more helpful than receiving generic nutritional advice. Nudge theory was first developed by Thaler and Sunstein [25]. Briefly, decisions about certain behaviors are made in a choice architecture that can be manipulated to favor a particular choice as the most likely outcome while maintaining freedom of choice. This *nudging* approach to messaging used in our study stands in contrast to a more restrictive system such as the banning of certain foods or the prohibition of alcohol or smoking. Daily personalized messages conveyed through the *GLOOK!* mobile app were intended to be advisory and, as much as possible, suggest positive choices rather than stimulating guilt over poor choices.

**Table 1:** Examples of feedback provided by physicians after reviewing the previous day’s data.

Participant number	Date	Physician feedback
2001	February 22, 2019	“Main issue is the high sugars after lunch and dinner, White flour-based bread and pizza base are causing problems, consider multigrain bread and a healthier choice for dinner. Good steps but no recorded activity. Try for 20 minutes extra exercise of moderate level per day.”
2001	February 23, 2019	“Good morning activity and low carbohydrate breakfast kept things nicely controlled thorough the morning. Low activity after lunch and multiple carbohydrate snacks in early afternoon kept blood sugar high in the afternoon. Try some low-GI snacks e.g. cheese on Vita Wheats or fruit (banana, berries etc.).”
2001	February 24, 2019	“Excellent morning after low carb breakfast. The coatings of schnitzels are a trap as they contain rapidly absorbed carbohydrate. Steamed chicken breast may have been a better choice. Well done for the extra exercise on the bike. Although exercise can acutely put the blood sugar up, the overall effect will be positive.”

The reviewed studies suggested that the design and development of diabetes self-management apps must be informed by an understanding of the needs and desires of the people who will use them and must incorporate the features and support mechanisms that patients value [11,12,15,17,26]. Many of the almost 2000 diabetes self-management apps available on the market do not discriminate between type 1 diabetes (T1D) and T2D, although studies have shown that people with T2D favor different app features than those with T1D. For example, in Australia, patients with T2D primarily use mobile apps for glucose monitoring, whereas patients with T1D use apps for carbohydrate counting [3]. This emphasizes the need for an individualized, customized app design.

### Goal of This Study

This clinical study at the Eastern Health Clinical School (Box Hill Hospital, Melbourne, Australia) aims to evaluate the satisfaction of patients with T2D with wearable technology alongside using a diabetes management app and examine the potential effectiveness of physicians’ real-time feedback in promoting behavior change around participants’ diet, activity, and health choices. The 12-day *GLOOK!* diabetes management system trial was designed as a pilot study for feasibility and proof of concept, with the primary goal of diabetes prevention.

Over a 5-month period in 2019, we aimed to test the hypothesis that wearable devices with real-time feedback from a physician might motivate behavior change in participants with T2D. The study used wearable sensor technology to track glucose profiles, medication, insulin dose, food and drink intake (through self-reported photographs of every meal), and activity levels of participants with T2D. Participants were provided with daily personalized SMS text message advice from a physician who had access to all the study participants’ collected data. A physician rather than a dietician reviewed the data and offered recommendations via the app as we monitored medication use and exercise, as well as diet.

## Methods

### System Design

We analyzed and identified the preferred features of a mobile app diabetes management system, as discussed in prior study. The analysis informed the design of the mobile app *GLOOK!*, which was designed and developed by an academic research team and tested at the Box Hill Hospital. The research team comprised endocrinologists, information technology and knowledge management experts, developers, interaction designers, and health specialists. The team was supported by diabetes nurse educators at the Eastern Health Clinical School.

**Figure 1:**
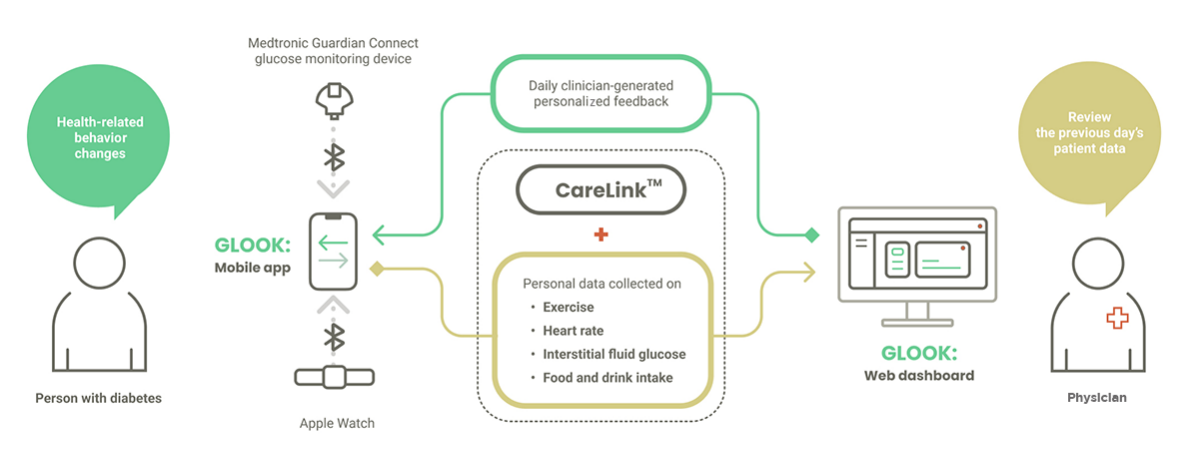
Schematic of the *GLOOK!* system connecting a patient to a physician.

*GLOOK!* was designed as a diary-style tracking and feedback-delivery system. The system integrates 3 components: a smartphone-based app for data collection and analysis, a smartwatch linked to the Apple Health App for activity recognition, and a wearable device—the Medtronic Guardian Connect (Medtronic Pty Ltd) continuous glucose monitor—for continuous interstitial fluid glucose monitoring. Although 2 of the 3 components were off the shelf, the third—the *GLOOK!* app that integrated all the data—was specifically designed and developed at an Australian university. In this paper, the app is referring to the *GLOOK!* app (Figure 1).

The system combines user input and sensor data to track patients’ behavior and food intake data and medication and insulin use and record patients’ daily activities. Other personal data were tracked using the mobile device’s built-in sensors; these included insulin use, number of steps, heart rate, and glucose levels. The uploaded data were integrated into the smartphone app, which also enabled participants to record their activities and food intake. The study physician received a report on these integrated data, which allowed the creation of personalized feedback for each study participant.

### User Interface Design

**Figure 2:**
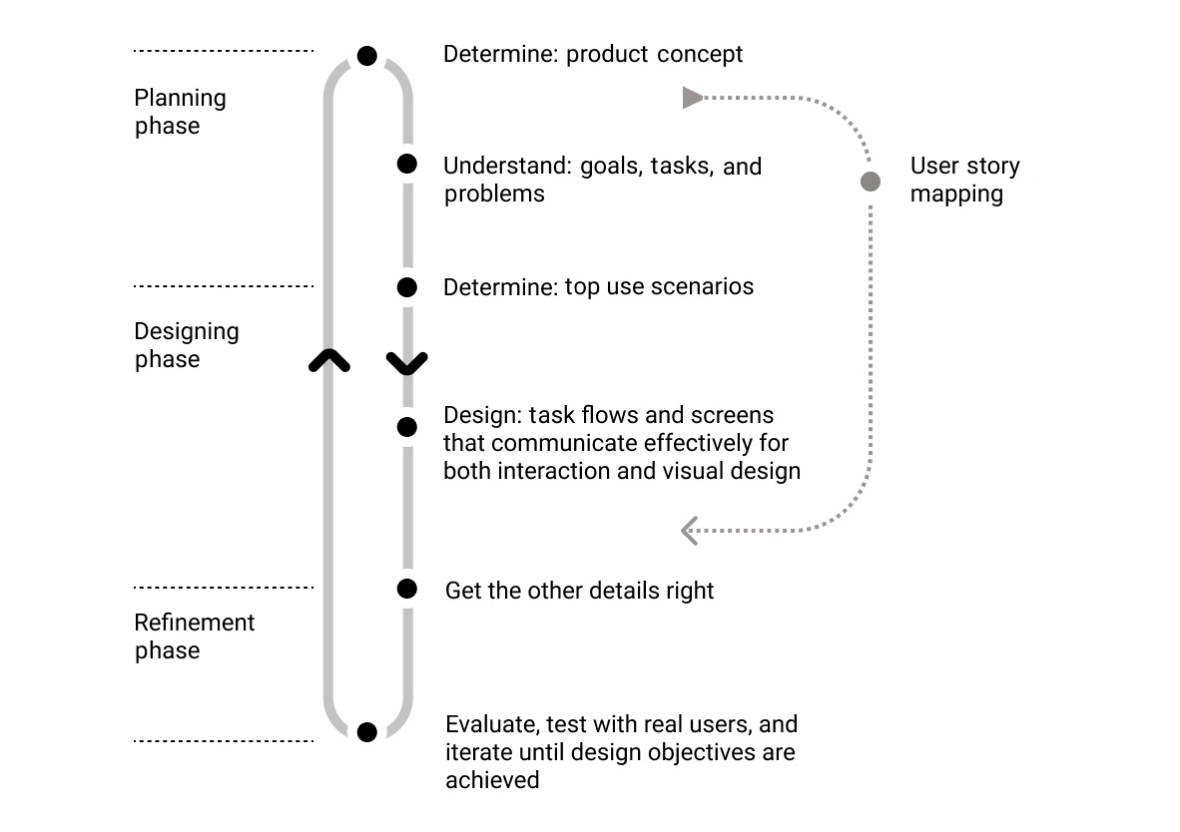
Communication-driven design process applied in the *GLOOK!* app’s graphical user interface design.

The *GLOOK!* mobile app’s graphical user interface uses a communication-driven design process to simulate intuitive communication between patients with T2D and physicians. User experience expert Everett McKay [27] regards the user interface as a mode of conversation between users and technology. The user interface enacts tasks so that users can achieve their goals through the language of the user interface instead of natural language. The communication-driven design process (Figure 2) underpins a clear understanding of users’ needs, tasks, and goals. The top-prioritized needs, tasks, and goals for both patient and physician user groups in the design of the *GLOOK!* app were determined using a user story mapping method.

### Empirical User Study

**Figure 3:**
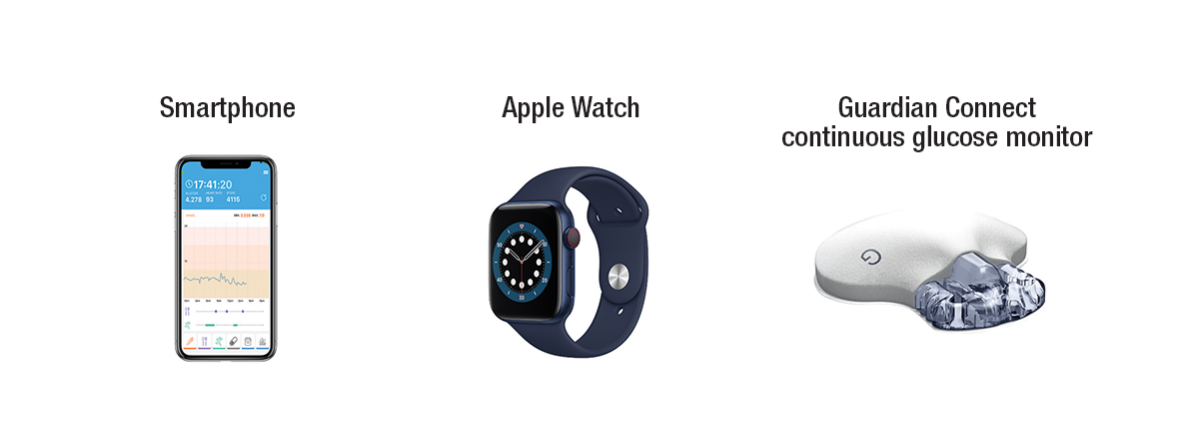
Technology used in the *GLOOK!* system.

A technology package was prototyped for the study, assembling 3 applications to gather data and provide 1-way communication between the physician and participants (Figure 3).

**Figure 4:**
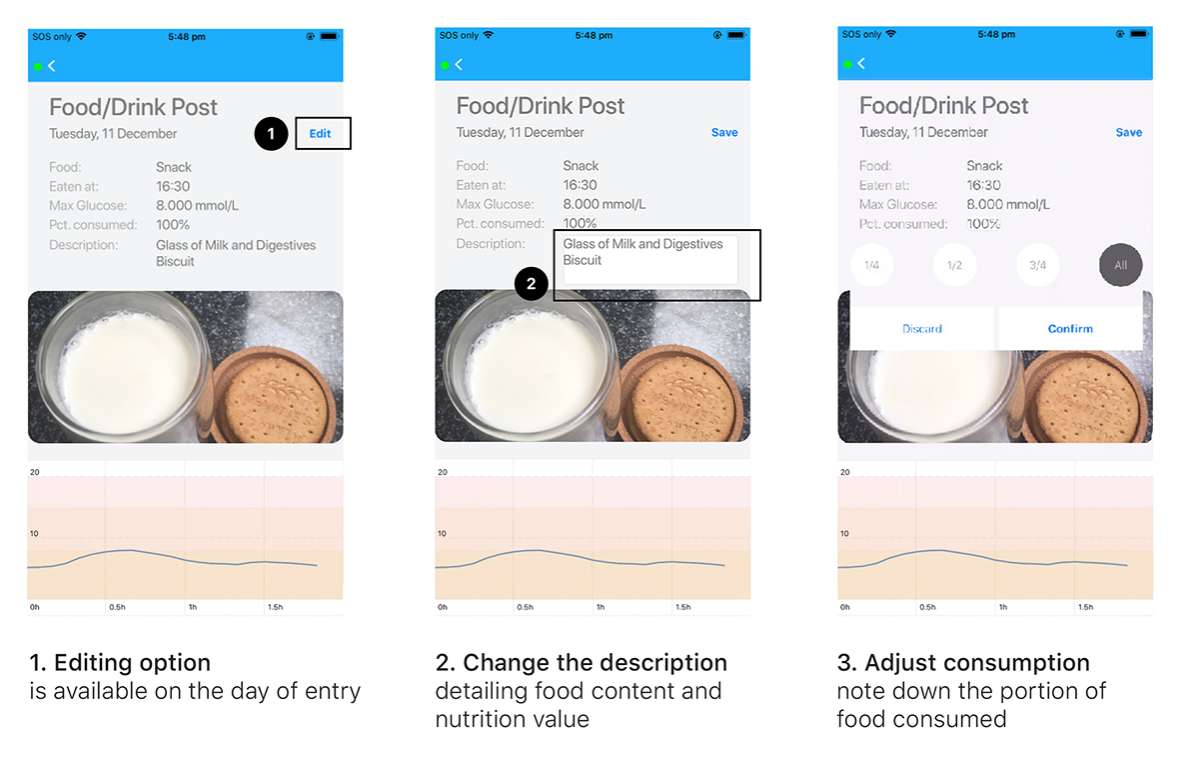
Examples of *GLOOK!* app screens for uploading and editing food and drink images.

The iOS app (*GLOOK!* app developed by Monash University) was installed on an Apple iPhone provided to the study participants. Participants also wore an Apple Watch linked to phone-recorded data on heart rate and steps. The Medtronic Guardian Connect continuous glucose monitor was applied to the skin according to the manufacturer’s instructions, and it provided 24-hour continuous glucose monitoring for 6 days. At the end of 6 days, another continuous glucose monitor was applied, thus providing a total of 12 days of data. The glucose data were *scrubbed* from the CareLink website. All sensor data were combined for display in the *GLOOK!* app (Figure 4). The app enabled participants to self-record activities and planned exercise episodes, as well as record all meals and snacks by inputting text descriptions and photographs.

All data in the app were mirrored on a webpage that was accessed by the physician, who reviewed the previous day’s data each morning and provided text-based feedback, which would appear both in the app and as a notification. There were no opportunities for 2-way communication with the physician. Feedback was limited to 2 to 3 sentences and concentrated on a few aspects of health-related behavior, with the aim of providing positive recommendations for improvements. Examples of daily feedback responses are listed in Table 1.

### Recruitment

A total of 15 patients with T2D were recruited for the pilot study from a larger cohort of registered outpatients attending a large public hospital in Melbourne, Australia.

**Figure 5:**
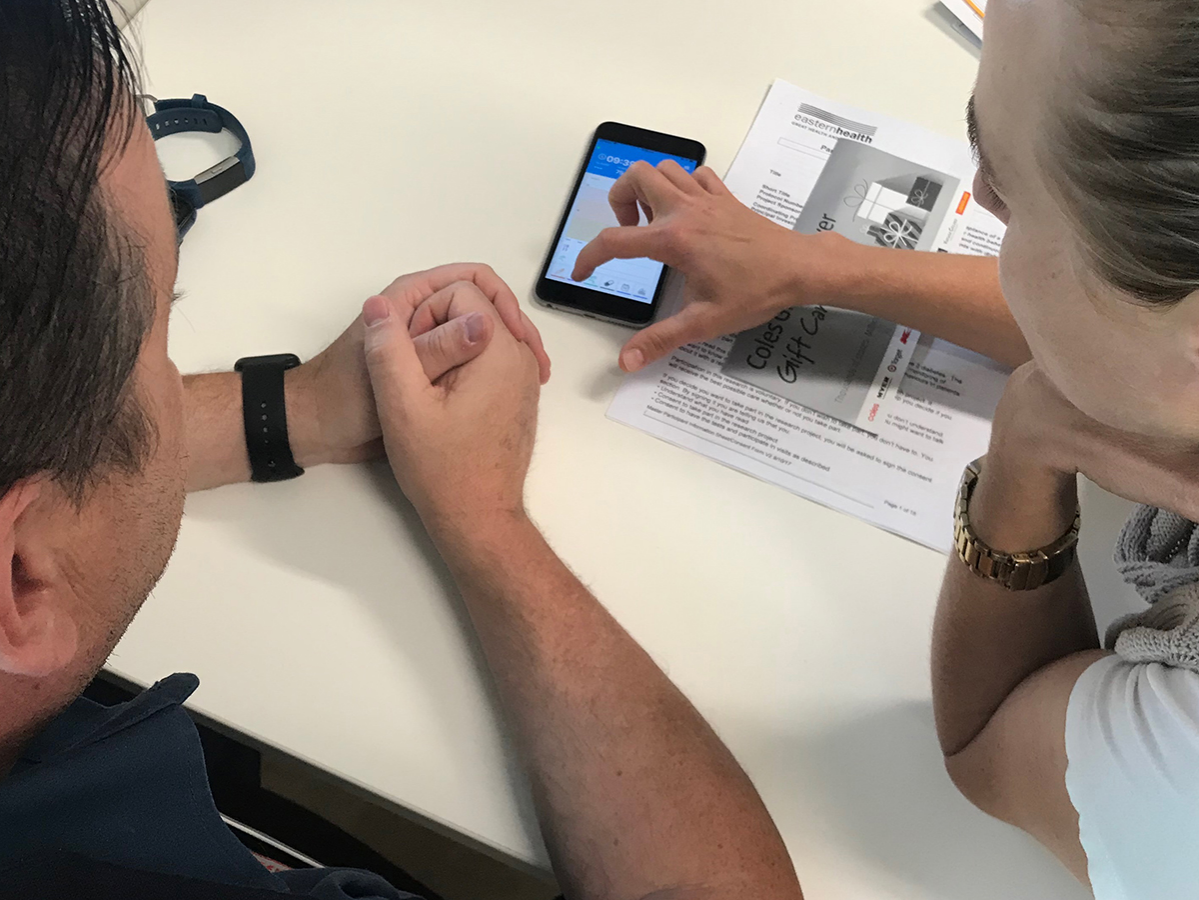
Study participant being taken through the *GLOOK!* app functions.

Participants were preselected from the public hospital database and associated clinics based on their T2D diagnosis (none were recently diagnosed), basic computer skills, and access to digital media. In addition, the recruited participants had no disabilities or health conditions that could interfere with their activity levels. Of the 15 participants, there were 4 (27%) women and 11 (73%) men. Patient ages ranged from 42 to 65 years. Participants aged >65 years were excluded because of presumed unfamiliarity with the use of smartwatches, smartphones, and SMS text message communications. It was important that participants had a reasonable familiarity with using smart technology (smartphones) and the ability to adapt to any smart gadgets and devices provided in this study (Figure 5).

### Qualitative Design Research Methodology

#### Overview

Two Monash University design researchers conducted and audio recorded 30 in-depth, semistructured, and descriptive interviews with the 15 study participants. The interviews were conducted twice over the period of the 12-day study: once at the beginning—while participants were being fitted with the sensor and trained on *GLOOK!* app use—to provide a baseline and then again at the end of the study.

Interview questions were based on the study’s primary outcome measures and covered the participants’ background, how they managed their diabetes before and after the study, their digital literacy, their attitudes toward managing their health and diet, and their levels of satisfaction with using digital eHealth technology represented by the *GLOOK!* app. The questions acted as open-ended prompts for discussions. Design research methods used a conversational interview technique that encouraged participants to offer personal narratives and describe their lived experiences [28].

#### Power Dynamics

Although sourced from public clinics, only 1 patient was known to the lead physician of the study before the study. The feedback was anonymous, and the physician was not identified.

#### Data Analysis

Patient characteristics were compared from baseline to the final visit using the Student *t* test (2-sided with equal variance). In addition, activity levels (steps), average blood sugar, glucose variability, glucose time in range, and resting pulse rate were compared for the first 4 days of the study with those for the final 4 days of the study using the mean of 4 days’ data for each participant compared by Student *t* tests. Statistical analysis of the collected biometric data was performed using SPSS software (IBM Corporation).

All audio recordings from participant interviews were reviewed by 2 design researchers using a deductive framework approach for data analysis [29,30]. A Microsoft Excel spreadsheet was developed for the thematic synthesis of interview data, where themes were drawn from the study’s hypotheses and aims. Interview data were abstracted, synthesized, and then charted according to the parts of the framework they were related to. The tabular form allowed a snapshot of insights and keyword searches. Novel themes that did not correspond directly to the study aims or objectives but were identified during the framework analysis were added during the process.

### Ethics Approval

Ethical approval for this pilot project was applied for and was granted (project ID LR63/2017) by the Eastern Health Research Ethics committee on October 9, 2017.

## Results

### Clinical Outcomes

Our study’s participants were on a wide range of antidiabetes medications (Tables 2 and 3), and these were not changed during the 2 weeks of the study apart from variations in insulin doses at the patient’s discretion. Specific advice regarding medication changes was not provided in the feedback.

**Table 2:** Glucose lowering therapies at baseline (n=15).

Therapy	Participants, n (%)
Diet alone	0 (0)
Insulin	2 (13)
Metformin	14 (93)
SU^a^	2 (13)
TZD^b^	0 (0)
DPP-4^c^	1 (7)
GLP-1^d^	6 (40)
SGLT-2^e^	3 (20)

^a^SU: sulfonylurea.

^b^TZD: thiazolidinedione.

^c^DPP-4: dipeptidyl peptidase-4 inhibitor.

^d^GLP-1: glucagon-like peptide-1 agonist.

^e^SGLT-2: sodium-glucose cotransporter–2 inhibitor.

**Table 3:** Baseline characteristics of the patients in the study (n=15)^a^.

Characteristic			Values, mean (SD; range)
Age (years)			54.07 (7.16; 41-65)
Height (cm)			135.67 (12.44; 110-158)
Weight (kg)			98.09 (10.50; 80.8-115.6)
Systolic BP^b^ (mm Hg)			135.67 (12.44; 110-158)
Diastolic BP (mm Hg)			85.07 (9.11; 71-103)
BMI (kg/m^2^)			31.95 (3.64; 26.0-40.1)
Waist to hip ratio (n=13)			0.98 (0.04; 0.91-1.06)
	HbA_1c_^c^ (%)	7.94 (2.14; 5.8-13.6)	
	HbA_1c_ (mmol/mol)	63.3 (23.4; 40-125)	
Fructosamine (mmol/L)			295.80 (80.78; 221-545)
Total cholesterol (mmol/L)			4.78 (0.89; 3.7-6.70)
HDL^d^ cholesterol (mmol/L)			2.39 (1.06; 0.90-4.70)
Triglycerides (mmol/L)			1.19 (0.22; 0.90-1.87)
Creatinine (µmol/L)			82.27 (34.30; 39-163)

^a^Female to male ratio was 4:11.

^b^BP: blood pressure.

^c^HbA_1c_: hemoglobin A_1c_.

^d^HDL: high-density lipoprotein.

Electronic data collected by wearable arrays were incomplete for a variety of technical reasons. The original data set contained 30,000 data points for each patient. It was estimated that approximately 79.86% (2300/2880) of the glucose trace and 76.19% (4320/5670) of the activity and pulse rate data were available for analysis. There were sufficient data for a feedback response on 83% (10/12) of the days of the study. As a pilot or feasibility study, this study was underpowered to detect changes in HbA_1c_, nor did it have a control group that did not receive feedback.

The effects of the intervention on anthropometry and parameters derived from bioelectrical impedance are shown in Table 4. Body weight fell 0.65 kg on average (from 98.1 kg to 97.45 kg). This failed to reach significance, with *P*=.06. There were also falls in systolic blood pressure, diastolic blood pressure (4.47 mm Hg and 2.93 mm Hg, respectively), and heart rate by 1.67 beats per minute; however, these did not reach significance. There were no significant changes in waist circumference and waist to hip ratio. Bioelectrical impedance analysis revealed falls in both lean mass and fat mass, with the fat mass decline exceeding the lean mass decline (3.47 kg vs 0.57 kg). None of these changes or changes in other bioelectrical impedance parameters reached significance in this small study.

**Table 4:** Changes in measured parameters from day 1 to day 12 of the study.

Parameter	Mean change from day 1 to day 12	*P* value
Systolic BP^a^ (mm Hg)	−4.47	.21
Diastolic BP (mm Hg)	−2.93	.09
Heart rate (beats/min)	−1.67	.41
Weight (kg)	−0.64	.06
BMI (kg/m^2^)	−0.91	.12
Waist hip ratio	0.01	.27
HbA_1c_^b^ (%)	−0.22	.004
Fructosamine (mmol/L)	−10.36	.16
Creatinine (μmol/L)	3.27	.10
Total cholesterol (mmol/L)	−0.25	.15
LDL^c^ cholesterol (mmol/L)	−0.15	.30
Triglycerides (mmol/L)	−0.24	.43
HDL^d^ cholesterol (mmol/L)	0.01	.66

^a^BP: blood pressure.

^b^HbA_1c_: hemoglobin A_1c_.

^c^LDL: low-density lipoprotein.

^d^HDL: high-density lipoprotein.

The changes in blood parameters and vital signs between the baseline and final visits are shown in Table 4. HbA_1c_ fell by 0.22% (*P*=.004). Fructosamine fell by 10.36 mmol/L; however, this did not reach statistical significance. There were favorable movements down in total cholesterol, triglycerides, and low-density lipoprotein cholesterol and favorable movements up in high-density lipoprotein cholesterol, but none of these changes reached statistical significance.

Activity was assessed by the number of steps per hour, as measured by the Apple Watch. The average number of steps per hour declined from 442 in the first 4 days to 399 in the last 4 days of the study, and this did not reach significance. Heart rate, as measured by the Apple Watch, was analyzed for changes in maximum heart rate, SD of heart rate, and resting heart rate (defined as heart rate at 5 AM), comparing values for the first 4 days of the intervention with those for the last 4 days of the intervention; no significant changes were found.

**Figure 6:**
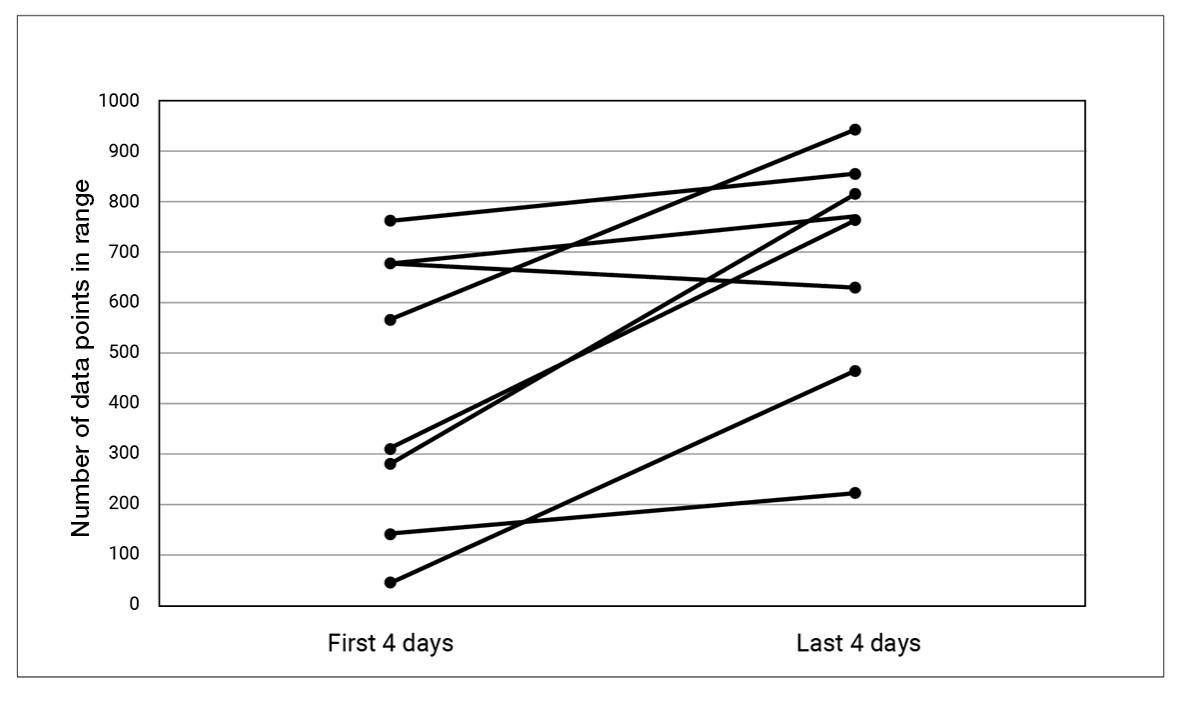
Mean interstitial glucose recorded over the first 4 days of the study compared with last 4 days of the study.

Changes in the continuous blood glucose trace were examined from the first 4 days of the study (days 1-4) and compared with those for the last 4 days of the study (days 9-12; Figure 6). There was no change in average blood glucose (9.18 vs 9.15 mmol/L; *P* value not significant). There was no change in glucose variability, as measured by SD (1.90 vs 1.76; *P* value not significant). There was no change in time in the range defined as the number of glucose data points ≥4.0 mmol/L and <10 mmol/L (636 data points vs 736 data points; *P*=.16). Of the 15 participants, 1 (7%) participant had no data points in the range, and 2 (13%) participants had all data points in the range. If very poorly controlled or very well-controlled patients are removed from the analysis, then there is a significant improvement in the time in range.

### Interview Outcomes

#### Overview

Analysis of the interviews showed that participants were curious about their personal health information and were keen to learn how to use real-time tracked information to manage their health. Although they tended to have a long-standing relationship with their family physician, they felt that the depth of information about their diabetes from scheduled general practitioner (GP) checkups was limited. Most participants planned their meals as per family and convenience rather than nutrition, influencing meal choices and quantities. Activity levels varied; fewer than half engaged in planned exercise, and only 13% (2/15) were *high-level* exercisers. Almost all participants expressed general satisfaction with the study. They felt that they had learned something about how their dietary habits, in particular, had affected their glucose levels. They appreciated having to be accountable to the physician providing them with daily feedback but would only want to continue using the *GLOOK!* system if the usability and reliability of the app were improved.

#### Results From Specific Domains in the Study

##### Experience With Digital Health Technology

Of the 15 participants, 6 (40%) had previously used health-tracking devices. Of these 15 participants, 10 (66%) had used smartphone apps before for monitoring their health, and 11 (73%) had sought additional information from the internet during the study period.

##### Engagement With Traditional Health Care

All patients reported having a relationship with their existing family GP for a long duration (between 2 and 30 years), with an average of 12 years. Adherence to regular health practitioner visits varied; almost half (7/15, 47%) made 3-monthly GP visits for prescription renewal or checkups. Many had been referred to dieticians and other allied health professionals but did not attend regularly after the initial education following a diabetes diagnosis. Only 20% (3/15) of patients visited their diabetes nurse educator at either the 3- or 6-month intervals. Patients who saw their GP more regularly visited their diabetes nurse educator more frequently. Patients felt that the usefulness and amount of advice and follow-up on diabetes from health professionals varied. One of the study’s patients who initially saw a diabetes nurse educator felt that “there was nothing that she could tell me that I didn’t already know” (P2014). Another reported that their physician did not give advice on aspects of diabetes management: “He’s a doctor, not a physical educator” (P2002).

A patient who visited their GP strictly for prescription renewal suggested a strong desire to be given relevant information about T2D self-management: “...don’t tell me that I’m a naughty boy and that I’m sick but tell me how to manage it” (P2009).

Patients we talked with showed curiosity and willingness to learn even in the perceived absence of professional health advice: “I’m trying to figure this stuff out on my own” (P2010).

One of the patients expressed exasperation at having to deal with a chronic disease with so many individualized variables:

I’ve been diabetic for a bloody long time. Twenty-odd years. And I still don’t get it...I just found the whole process damn confusing.

P2007

##### Diet

Of the 15 patients, 11 (73%) cooked for themselves at least part of the time. For many, family requirements restricted their free choice of meals. Some noted a lack of time, not wanting to plan, or simply finding it *easier* to eat out. Convenience outweighed nutrition in meal choice, and most felt that their diet could be improved, that it was “not the best, but not the worst” (P2001).

##### Exercise

Only 27% (4/15) of the participants actively engaged in moderate planned exercise and only 13% (2/15) in high-level exercise. One of the participants felt that *GLOOK!* study had encouraged them to engage in more planned exercise; they stated that the study physician’s feedback around exercise was personalized and empathetic in its delivery:

So he’s like, “can you see if you can just increase a little bit?” and suggested a five-minute walk. And I’m thinking, well, he understood that I do have issues. And he’s like, “We’re not asking you to go for a 10-kilometer run”

P2007

##### Satisfaction With the GLOOK! App

Of the 15 participants, 13 (87%) said they would have been happy to continue using the *GLOOK!* app and felt that using it for longer would have enabled them to gain a better understanding of patterns in their personal data. Of the 15 participants, 2 (13%) said they would not continue using the *GLOOK!* app as they felt the wearable Guardian Connect device was too invasive. Specific comments regarding the usability of the app included the need for faster uploads, a visible icon indicating that the app was processing data, and the ability to zoom in on the data displays. Other suggested features were comparative visuals of what was *normal* for people without T2D and how the user’s levels compared, weekly summaries, and highlights and *next steps* to increase motivation.

Some participants felt that photographing food was difficult to do in public, especially when dining out. From feeling self-conscious to feeling as if it was a nuisance, participants noted that this aspect of the study was the biggest burden. However, at the same time, participants commented that photographing their food made them feel more accountable (to themselves) and aware of what they were eating and drinking. Participants would have liked 2-way communication with the physician, especially to clarify aspects of the meal they had photographed or to ask a question about the SMS text message feedback:

It would have been nice if there was an option to be able to respond to the feedback and ask questions, because...he [the physician responding] gives you a direct, “if you do this, this and this....” But that was based on assumptions. Like, for example, yesterday my lunch was a quiche where it’s not a real egg and bacon pie. He’s like, “Your sugar spiked because of the crust on that.” But it doesn’t have a crust on it because I made it myself. It’s just basically egg and bacon in a pie dish. So just to give back and say, “Well, you actually haven’t quite got the advice right”...at the moment it’s sort of a one-way street.

P2001

##### Satisfaction With the Feedback

All participants were satisfied with the tone and helpfulness of the feedback, even when the physician’s comments on food choices were not always positive: “It’s nice having someone in your corner” (P2015).

They looked forward to receiving the feedback and appreciated the personalized aspects. Some perceived that the information was already known to them; it validated their own knowledge about their diet and habits as a patient suggested:

...it didn’t provide me anything I didn’t know...I might not have known it at the front of my mind, but I DO know it

P2004

Others gained useful new insights and felt more in control of their choices.

Patient 2015 felt more in control and could see how continued app use might improve knowledge of their specific health behavior: “If I’d known what I know now, then things would be so much better” (P2015).

##### Behavior Change

Of the 15 participants, 3 (20%) noted an increase in their feelings of positivity and well-being following the study, and some felt that participation increased discussions and changed family routines around healthy food choices. Others determined that they would measure glucose levels daily rather than every few days as a result of being in the study. An awareness of the need to change behavior by acting upon the advice was suggested: “It’s like anything, if you’re getting the information, it’s worth nothing if you don’t work off it” (P2001).

Of the 15 participants, 2 (13%) participants mentioned feeling supported and motivated to change their behavior as a result of having the physician’s feedback on a daily basis, as patient 2007 commented the following:

All this, I consider learning; it’s a learning thing. And it’s understanding. For diabetes is massive. And I mean, even this—being held accountable. And I think, as I said, my diabetes people are fantastic. But they can’t phone me daily.

P2007

## Discussion

### Principal Findings

This pilot study used wearable technology to gather data on activity, exercise, pulse rate, interstitial fluid glucose, and food intake and give patients with T2D daily text-based feedback that would provide short advisory comments (nudges) on food intake and activity based on the previous day’s data.

Advice on behavior change is often based on the average responses of groups to particular foods rather than on individual responses. It has recently become clear that there are large differences between individual glycemic responses to food and that approaches based on average responses, such as a glycemic index, may be inherently flawed [31]. Therefore, a system that uses individual glycemic responses as the basis of dietary recommendations is appealing.

It is crucial that new technologies are brought to bear to facilitate behavior change both through providing real-time visibility of blood glucose profiles and by providing nudging messages to reinforce positive messages on a frequent (daily) basis. We believe that regular, frequent, positive, and suggestive feedback using the strategy of nudging will, over time, significantly modify behavior and prevent the development of diabetes and reduce other cardiovascular risk factors such as hypertension and hypercholesterolemia, thus leading to weight loss or a positive change in body fatness.

### Comparison With Prior Work

A survey of Australian patients with T2D about a *perfect* diabetes self-management app [11] identified personalization and the ability to monitor information about sugar levels and medications over the long term as the most desired features. Baptista et al [11] also suggested that people want the app to address the psychological, cognitive, and emotional aspects of living with diabetes, as well as assist with the practical elements of diabetes management. We also acknowledge several other empirical studies that have investigated the perceptions of patients with diabetes and the potential for using mobile health apps for behavior change and improved health outcomes. Several studies [6,32-34] found a high degree of patient satisfaction with receiving SMS text messages (whether motivational or educational) and that patients found this useful and beneficial. In their study, Dobson et al [33] noted that there was a significant difference in perceptions of being supported between patients in the mobile app intervention group that received generic SMS text messages and the nonintervention control group that did not receive SMS text messages.

The main difference between our research and others is in testing the usefulness of the *GLOOK!* app as a complementary tool for continuous communication between physicians and patients with T2D. This demonstrates that there is a certain gain in reinforcing positive behavioral changes through timely and personalized mobile phone messages sent on a daily basis.

Although the optimum frequency of messages is not yet known, our study and others [5,6,24,32,33] suggest that regular text-based feedback can increase patient motivation and understanding of how their diet and activity can affect T2D. Previous research on the use of diabetes self-management applications has shown increased efficacy in interventions that allow more patient customization and choice over the frequency of text-based message delivery [6]. The timing of received messages can also influence patient acceptance of and adherence to the program; enabling patient choice over what time of day they receive messages reduces the effect of them feeling *nagged* and may allow more reflection and interpretation [5]. An ideal protocol may comprise a personalized text-based message per day at a time chosen by the patient, with the option of receiving additional generic messages related to diabetes education and health.

The diabetes self-management app studies we reviewed deployed both generic (not based on individual data inputs) and personalized tailored messages. The degree of personalization varied. On one end, Dobson et al [33] used SMS text messages conveying daily reminders (eg, to check glucose levels), reassurance and praise, tips for diabetes care, and diet to determine whether these might affect a change in HbA_1c_ levels or any positive behavior change in patients with poorly controlled diabetes. These messages were not triggered by continuous glucose monitoring or other (eg, activity) inputs, and the only personalization was the recipient’s name. On the other end, Park et al [24] used similar data inputs as used in the *GLOOK!* study to trigger messages customized in response to data inputs.

In our *GLOOK!* pilot study, we found that the coaching aspect of having the physician assess an individual’s data and share their feedback through SMS text messages was a key driver of motivation and emotional support. One of the study’s participants shared the following:

I like having someone to report to. I find it keeps me on track. It keeps me honest; it keeps me motivated

P2007

Although it is possible that participants might become *addicted* to this level and type of feedback, similar to any other coaching-type interaction, the frequency and extent of feedback could be scaled back as the patient develops self-efficacy and “...an understanding of what is going on and even where you can improve in things as a real-life daily action” (P2007). In the *GLOOK!* pilot study, patients’ average steps declined over the study period, suggesting that the effect of the intervention was wearing off over the 2-week study. Furthermore, patients reported in the interviews that their motivation to exercise fluctuated according to their mood, mental health, and social situation. For sustained behavior change, methods for enhancing motivation and increasing activity levels over the long term would need to be tested.

### Limitations

The study had some limitations because of limited resources, including time and specific technical components (eg, Medtronic continuous glucose monitor, Apple Watch, and Android smartphone) used for the *GLOOK!* app implementation. This explains the relatively short period (12 days) that the patients used the system for the physician to communicate with them. A small group of 15 patients was recruited, as the pilot study objective was to collect initial feedback on the new treatment process and demonstrate the feasibility of the technology to communicate personalized feedback rather than a full clinical trial. This also explains the lack of a control group in the study design, which is another limitation. One patient had very poor control, with very high blood glucose levels; they needed a treatment change, which was not performed in the context of this study. In retrospect, we would have selected patients with intermediate levels of control.

The study was intended to address the consumption of foods with a high glycemic index rather than a poor diet in general (eg, one high in saturated fat and sodium); this is a limitation of the study. The tool had a basic functionality to support glucose levels, activity data monitoring, and physician feedback. Feedback from the participants regarding their experience using the *GLOOK*! app corresponds to some of the limitations and will be addressed in the planned new phase of the study.

### Future Research

Despite the small sample sizes in our pilot study, our findings support the potential of mobile app–based, daily personalized physician feedback as an intervention for positive changes in behavior and health outcomes in people with T2D. A follow-up study is needed to ascertain both the long-term engagement with the app and the extent to which long-term behavior change is feasible. Specific areas to be explored in follow-up studies include the following:

- How feedback that is analyzed and delivered via AI rather than by a physician might reproduce the personal and motivational effect of coaching-style feedback
- Whether 2-way communication enhances motivation for sustained behavior change
- How behavior change models could be deployed to personalize feedback messaging [5,34]

Interviews with patients with T2D in this study provided important insights into how the experience could be made more engaging and presumably more effective. Future wide-scale applications of daily personalized feedback delivered through an app would be limited by the availability of physicians or even specially trained dieticians to provide this feedback. This factor, and the learning points from our pilot study, could be incorporated in the design of new integrated wearable technology that will enable scaling up of this kind of intervention through the use of newer and less invasive sensor technology, possibly by deploying an AI approach in the generation of feedback. This approach aligns with predicted advances in customized diabetes treatment by incorporating machine-based algorithms [13,20,24]. The increasing capability of big data analytics could even generate personalized interventions for behavior change tailored to different patients’ needs for specific motivational techniques [5]. New technology will allow sufficient upscaling of this approach to have an impact on the incidence and community costs of diabetes.

### Conclusions

Our study suggests that providing daily physician-generated personalized feedback based on wearable sensor information and recorded food intake and activity data can produce favorable changes in glycemic and cardiovascular risk parameters even in the short term. The participants found the experience acceptable, and it provided them opportunities for positive long-term behavior changes. We plan to address the feedback collected through the interviews to redevelop the system and conduct a longer study with more participants.

## Abbreviations

AI: artificial intelligence
GP: general practitioner
HbA_1c_: hemoglobin A1c
T1D: type 1 diabetes
T2D: type 2 diabetes
